# White Bile Detected During Endoscopic Retrograde Cholangiopancreatography: A Rare Phenomenon and Its Clinical Implications

**DOI:** 10.5152/tjg.2025.25018

**Published:** 2025-03-19

**Authors:** İdris Kurt, Yalçın Taymez, Görkem Karadağ

**Affiliations:** Department of Gastroenterology, Trakya University School of Medicine, Edirne, Türkiye

Dear Editor,

An 87-year-old female patient presented with 1 month of jaundice and intermittent abdominal pain. She had experienced a weight loss of approximately 15 kg over 6 months. Her medical history included hypertension, diabetes mellitus, and atrial fibrillation. On physical examination, jaundice was noted along with a palpable, painless hydropic gallbladder (Courvoisier–Terrier sign) and excoriation marks on the skin due to itching. Laboratory tests revealed total bilirubin of 16.5 mg/dL, direct bilirubin of 13.9 mg/dL, alkaline phosphatase of 330 U/L, gamma-glutamyl transferase of 930 U/L, alanine aminotransferase of 125 U/L, aspartate aminotransferase of 107 U/L, C-reactive protein of 17.8 mg/L, and a white blood cell count of 5.6 × 10^3^/µL. Cross-sectional imaging demonstrated a mass lesion causing dilation of the intrahepatic bile ducts, the common bile duct, and the pancreatic duct (double duct sign) (Figure 1A). Radiologically, the patient was diagnosed with an unresectable pancreatic mass. Endoscopic retrograde cholangiopancreatography (ERCP) was performed for drainage, and endoscopic ultrasonography was performed for diagnostic purposes. During ERCP, following cannulation and stent placement, a large volume of colorless bile was observed (Figure 1B). Within several days, her bilirubin levels and cholestatic enzymes normalized. Histopathological examination revealed pancreatic adenocarcinoma. Since the patient did not meet the criteria for surgical operability, she was referred to medical oncology for systemic chemotherapy.

Colorless or “white” bile is a fluid lacking pigment, bile acids, and cholesterol, typically associated with biliary obstruction. Although its precise etiology remains unknown, a potential mechanism for its colorless appearance may be continued mucus secretion by the biliary epithelium despite the cessation of bile flow.^1^ Early hypotheses suggested that its formation could be related to the absence or dysfunction of the gallbladder (e.g., cystic duct obstruction or stenosis above the cystic duct); however, subsequent studies failed to confirm this.^2^ Mechanical obstruction of either benign or malignant origin may lead to the development of white bile.^3^ Particularly for distal malignant obstructions, the most common causes are pancreatic carcinoma, cholangiocarcinoma, and metastatic tumors.^4^ Although its clinical significance is still debated, some studies have found that in cases of malignant obstruction, the presence of white bile is associated with more pronounced jaundice, an increased risk of cholangitis, and shorter survival.^3^^,^^5^ In the largest case series reported to date, a comparison revealed that among patients who did not undergo surgery and had normal bile, the median survival time was 9.1 months (95% CI: 1.1-55.2), whereas those with white bile had a significantly shorter median survival of 2.9 months (95% CI: 0.2-25.9) (*P *= .003). Based on these findings, white bile has been identified as an independent risk factor for patient survival.^6^ Accordingly, when clinicians encounter white bile, they should remain aware of the potential for increased cholangitis risk and shortened survival, and thus proceed more rapidly and cautiously. Informed consent was obtained from the patient.

## Figures and Tables

**Figure 1.**
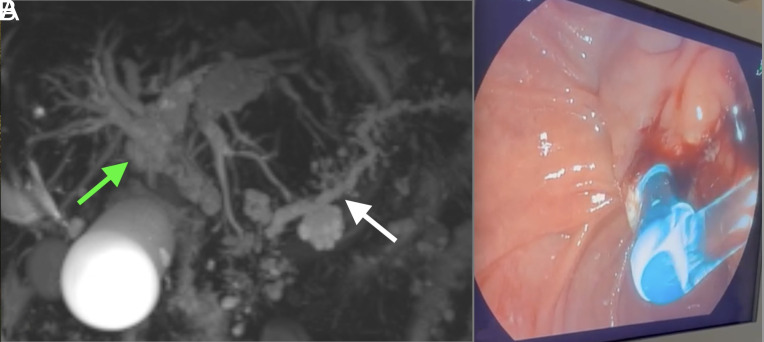
(A) The “double duct sign,” demonstrating dilation of both the biliary tree and pancreatic duct. (B) White bile observed draining through the newly placed plastic biliary stent.

## Data Availability

The data that support the findings of this study are available on request from the corresponding author.
